# Assessing Antigenic Drift of Seasonal Influenza A(H3N2) and A(H1N1)pdm09 Viruses

**DOI:** 10.1371/journal.pone.0139958

**Published:** 2015-10-06

**Authors:** Nipaporn Tewawong, Slinporn Prachayangprecha, Preeyaporn Vichiwattana, Sumeth Korkong, Sirapa Klinfueng, Sompong Vongpunsawad, Thanunrat Thongmee, Apiradee Theamboonlers, Yong Poovorawan

**Affiliations:** Center of Excellence in Clinical Virology, Faculty of Medicine, Chulalongkorn University, Bangkok, Thailand; Icahn School of Medicine at Mount Sinai, UNITED STATES

## Abstract

Under selective pressure from the host immune system, antigenic epitopes of influenza virus hemagglutinin (HA) have continually evolved to escape antibody recognition, termed antigenic drift. We analyzed the genomes of influenza A(H3N2) and A(H1N1)pdm09 virus strains circulating in Thailand between 2010 and 2014 and assessed how well the yearly vaccine strains recommended for the southern hemisphere matched them. We amplified and sequenced the HA gene of 120 A(H3N2) and 81 A(H1N1)pdm09 influenza virus samples obtained from respiratory specimens and calculated the perfect-match vaccine efficacy using the *p*_epitope_ model, which quantitated the antigenic drift in the dominant epitope of HA. Phylogenetic analysis of the A(H3N2) HA1 genes classified most strains into genetic clades 1, 3A, 3B, and 3C. The A(H3N2) strains from the 2013 and 2014 seasons showed very low to moderate vaccine efficacy and demonstrated antigenic drift from epitopes C and A to epitope B. Meanwhile, most A(H1N1)pdm09 strains from the 2012–2014 seasons belonged to genetic clades 6A, 6B, and 6C and displayed the dominant epitope mutations at epitopes B and E. Finally, the vaccine efficacy for A(H1N1)pdm09 (79.6–93.4%) was generally higher than that of A(H3N2). These findings further confirmed the accelerating antigenic drift of the circulating influenza A(H3N2) in recent years.

## Introduction

Influenza A virus is a major cause of acute respiratory disease in humans and is responsible for ~250,000–500,000 deaths annually worldwide [1]. Pandemic influenza A virus infection resulted in significant morbidity and mortality in 1918 (H1N1), 1957 (H2N2), 1968 (H3N2), and 2009 (H1N1) [2]. Subtypes of influenza A viruses are defined by the surface proteins hemagglutinin (HA) and neuraminidase, two major viral targets for the host immune system [3]. The HA protein of the influenza virus is cleaved by the protease enzyme in the host cells into two subunits: HA1 and HA2. The HA1 subunit plays a major role in binding to host receptor or neutralizing antibodies and represents major antigenic sites (defined as epitopes A, B, C, D, and E). In contrast, the HA2 subunit induces fusion of the viral envelope and endosomal host membrane [4]. The accumulation of amino acid mutations on the antigenic sites of HA1 reduces antibody recognition and drives antigenic drift [5–8].

The yearly updated trivalent influenza vaccine consists of inactivated virus with HA from types A(H1N1)pdm09 and A(H3N2), and one type B that best match the predicted circulating strains. Due to the high mutation rate of the influenza virus, however, typical vaccine efficacies are only around 50–60%, while complete protection against influenza-like illness is rarely achieved. An epidemiological study conducted in Thailand demonstrated an influenza vaccine efficacy among vaccinated children of 55–64% [9]. Therefore, epidemiological studies examining how antigenic drifts affect vaccine efficacy are required for an optimal vaccine design each flu season.

Towards assessing the influenza antigenic drift, differences between the vaccine strain and the circulating strain are quantified by the number of amino acid changes in the dominant epitope, a region on the HA1 protein recognized by the neutralizing antibodies. This *p*_epitope_ model has been used to estimate the antigenic distance for influenza virus and was shown to correlate with the vaccine efficacy to a greater degree than phylogenetic analyses or antisera hemagglutination inhibition assay [10]. To better understand the molecular evolution of influenza and assess how genetic drift affected vaccine efficacy, we examined the antigenic epitopes of HA1 of influenza A(H3N2) and A(H1N1)pdm09 circulating in Thailand from 2010 to 2014.

## Materials and Methods

### Specimen collection and preparation

Respiratory samples were obtained from Thai patients with influenza-like symptoms by the Center of Excellence in Clinical Virology at Chulalongkorn University. The inclusion criteria were fever (> 38°C) combined with respiratory symptoms such as cough, sore throat and runny nose. A total of 18,018 samples, collected from January 2010 to December 2014 in Thailand, were screened for influenza A and B virus by one-step multiplex real-time polymerase chain reaction (RT-PCR) and subtypes H1 and H3 were identified by specific primers as previously described [11, 12]. Part of the surveillance data has been previously reported [13, 14]. Samples tested positive for seasonal influenza A (H1 and H3) were then randomly selected for HA gene analysis.

All samples were stored anonymously and acquired with permission from the Director of King Chulalongkorn Memorial Hospital. The study protocol was approved by the Institutional Review Board, Faculty of Medicine, Chulalongkorn University (IRB No. 337/57) and the need for consent was waived because the samples were anonymous. This study was conducted according to the principles expressed in the Declaration of Helsinki and the IRB waived the need for consent because the samples were de-identified and anonymous.

### Nucleic acid extraction, PCR, and sequencing

Viral RNA was extracted using a commercial viral nucleic acid extraction kit (RBC Bioscience, New Taipei City, Taiwan) according to the manufacturer’s instructions. Viral RNA was transcribed into cDNA using the ImProm-II reverse transcription system (Promega, Madison, WI) and 1 μM universal 12 primers [11]. The HA sequences were amplified by PCR with the primer sets for human influenza A(H3N2) and A(H1N1)pdm09 virus (S1 Table). Briefly, a total reaction volume of 25 μl contained 10 μl of 2.5x mastermix (5Prime, Hamburg, Germany), 0.25 mM MgCl_2_, 0.5 μM each of forward and reverse primers, 2 μl of cDNA template and RNase-free H_2_O. The PCR parameters were 94°C for 3 minutes, followed by a total of 40 cycles of 94°C for 30 seconds, 55°C for 30 seconds, 72°C for 90 seconds, and 72°C for 7 minutes. PCR products were visualized on a 2% agarose gel and purified using the HiYield gel DNA fragment extraction kit (RBC Bioscience, New Taipei City, Taiwan). DNA sequencing was performed by First BASE Laboratories Sdn Bhd (Selangor, Malaysia).

### Nucleotide sequence accession numbers

The HA sequences of influenza A(H3N2) and A(H1N1)pdm09 obtained in the 2010 season in Thailand had previously been deposited in GenBank (S2 Table), while the HA sequences of the influenza A(H3N2) (in 2011–2014) and A(H1N1)pdm09 (in 2011–2014) isolates were submitted to GenBank under accession numbers KP335865 to KP335981 and KP941680 to KP941741, respectively. Moreover, the HA sequences of reference and southern hemisphere vaccine strains from influenza A(H3N2) and A(H1N1)pdm09 viruses included in phylogenetic analysis were obtained from the GenBank and GISAID databases. Their accession numbers were also included in the S2 Table.

### Phylogenetic analysis and antigenic characterization

The HA sequences were edited and assembled using SeqManPro (DNASTAR, Madison, WI). The ClustalX v.2.1 was used for the alignment of protein and nucleotide sequences [15]. The Akaike information criterion and maximum likelihood value indicated that the HKY+G model was the best fit model [16]. A phylogenetic tree of the HA1 coding nucleotide sequences was generated by Molecular Evolutionary Genetic Analysis (MEGA) version 6.06 [17] using a maximum likelihood tree by the HKY+G model with 1,000 bootstrap replicates; only bootstrap values over 50 were shown. The amino acid residues in the five epitopes (A—E) of A(H3N2) (A/Aichi/2/1968) and A(H1N1)pdm09 (A/California/04/2009) viruses were previously identified [18, 19]. The relative amino acid frequency in the epitope of HA1 was performed using WebLogo [20].

### Measurement of selection pressure

The selective pressure on encoding HA1 A(H3N2) and A(H1N1)pdm09 was examined by calculating the ratio of synonymous and non-synonymous substitutions (d*N*/d*S*, defined as *ω*) across lineage on a codon-by-codon basis. The individual site-specific selection pressure and *ω* were estimated using the single likelihood ancestor counting (SLAC) and fixed effects likelihood (FEL) methods contained in the HYPHY package [21]. All analyses utilized the Datamonkey online tool (http://www.datamonkey.org). The value of *ω* was estimated based on the neighbor-joining trees under the HKY85 substitution model. The significance level for a positively selected site by either SLAC/FEL or both methods was accepted at 0.1.

### Prediction of glycosylation sites

The NetNGlyc 1.0 server was used to predict potential *N*-linked glycosylation sites (amino acids Asn-X-Ser/Thr, whereby X is any amino acid except Asp or Pro) [22]. A threshold value of >0.5 for the average potential score suggests glycosylation.

### Estimation of vaccine efficacy using the *p*_epitope_ model

We estimated the vaccine efficacy of the influenza A(H3N2) and A(H1N1)pdm09 seasonal influenza viruses using the *p*_epitope_ method [7, 23–25]. Since the vaccine efficacy is linearly correlated with the antigenic distance between the vaccine strain and the dominant circulating strains, the antigenic distance, defined as *p*_epitope_, is calculated by the fraction of amino acid substitutions in the dominant HA epitope [10]. The association between vaccine efficacy and *p*_epitope_ is given by *E* = −2.47 × *p*_epitope_ + 0.47 for influenza A(H3N2) virus and by *E* = −1.19 × *p*_epitope_ + 0.53 for influenza A(H1N1)pdm09 virus [23]. The influenza A(H3N2) vaccine efficacy with *p*_epitope_ = 0 is 47% as a perfect match between vaccine and virus [10]. For the influenza A(H1N1)pdm09 virus, the vaccine efficacy is 53% when *p*_epitope_ = 0 [23].

## Results

### Phylogenetic analysis of A(H3N2)

Among the 18,018 respiratory samples of unknown etiology, 673 samples (3.7%) tested positive for influenza B and 3,034 samples (16.8%) tested positive for influenza A. The latter comprised 1,394 (46%) A(H3N2) and 1,640 (54%) A(H1N1)pdm09. From these, random selection resulted in the analysis of the HA gene from 120 A(H3N2) and 81 A(H1N1)pdm09 samples.

**Fig 1:**
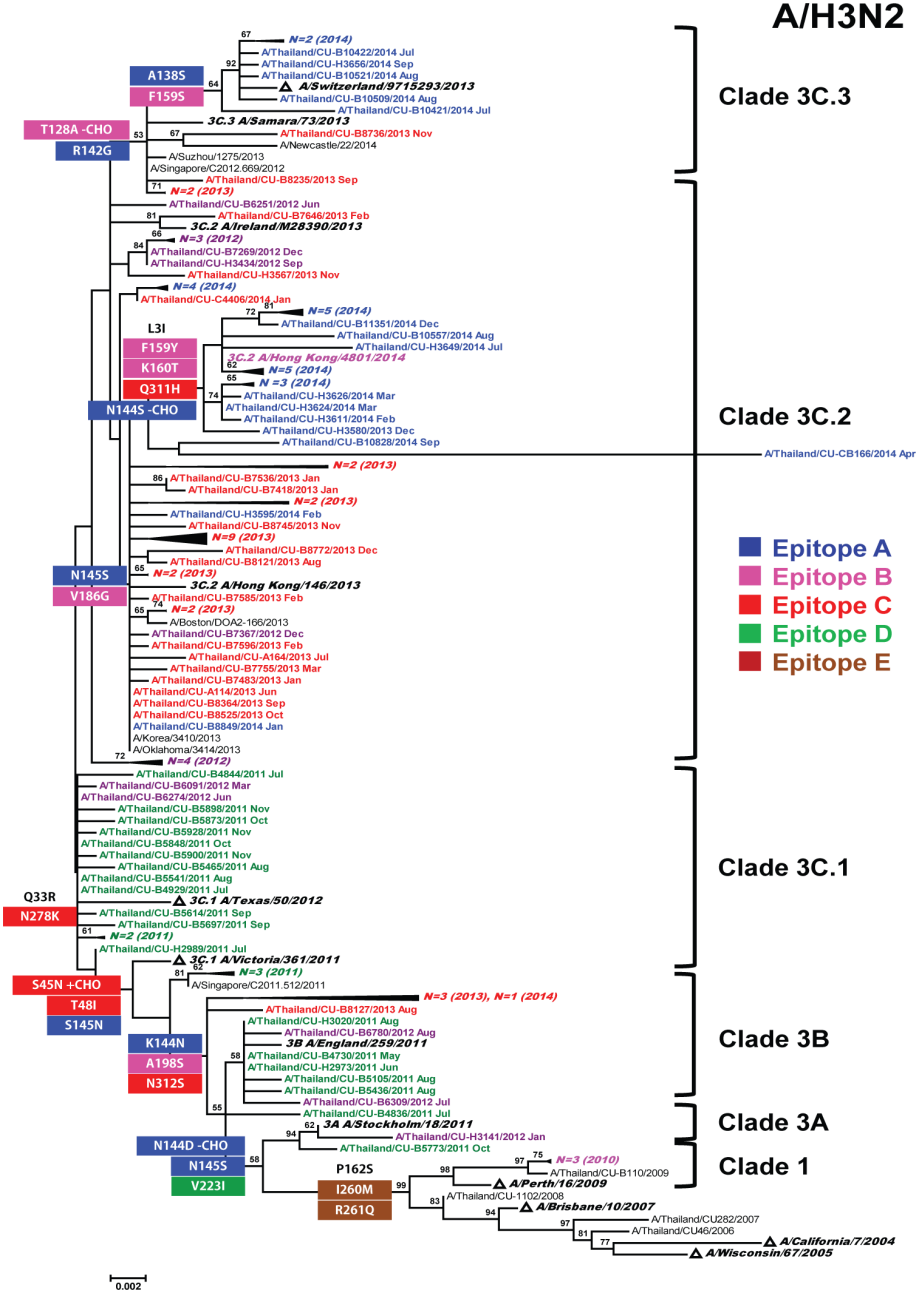
Phylogenetic analysis of HA1 nucleotide sequences of influenza A(H3N2). Sequences from 120 strains isolated in Thailand during 2010–2014 (designated A/Thailand/CU) were compared to the reference strains of known clades reported by WHO Influenza Center London (bolded) and the southern hemisphere vaccine strains recommended by WHO (denoted with triangles). The phylogenetic tree was generated by the maximum likelihood method using HKY+G model with 1,000 bootstrap replicates implemented in MEGA (version 6.06). Branch values >50 are indicated at the nodes. The signature amino acid changes to each clade are indicated in different colors by epitopes (A through E). CHO denotes site-specific glycosylation. Scale bar represents approximately 0.2% nucleotide difference between close relatives.

Comparison of the HA gene was performed on the A(H3N2) strains circulating during the 2010 (*N* = 3), 2011 (*N* = 24), 2012 (*N* = 16), 2013 (*N* = 41), and 2014 (*N* = 36) seasons and sequences from the southern hemisphere vaccine and reference strains. Phylogenetic analysis of the HA1 sequence showed that A(H3N2) strains from the 2010 season belonged to genetic clade 1 and shared amino acid substitutions at P162S, I260M, and R261Q (Fig 1). These 2010 strains clustered with A/Perth/16/2009, the reference vaccine strain for 2010, 2011, and 2012 (99.2% nucleotide and 98.9% amino acid identities). Meanwhile, the strains from the 2011 and 2012 seasons belonged to genetic clade 3 (3A, 3B, 3C.1, and 3C.2) and shared amino acid substitutions at N145S and V223I. Most strains (57.5%) belonged to sub-clade 3C.1 as defined by Q33R and N278K when compared to A/Victoria/361/2011, a vaccine strain for 2013 (S3 Table). The A(H3N2) strains from the 2013 and 2014 seasons grouped into clades 3C.2 and 3C.3. Most sub-clade 3C.2 strains (N = 66, 85.7%) possessed N145S and V186G compared to the A/Victoria/361/2011, the reference vaccine strain for 2013. In contrast, sub-clade 3C.3 was characterized by T128A, A138S, R142G, and F159S compared to A/Victoria/361/2011 (vaccine strain for 2013) and A/Texas/50/2012 (vaccine strain for 2014).

The overall HA1 nucleotide identities among the A(H3N2) strains compared to the given vaccine strains over the period examined were >97%, while the amino acid identities were >96% (Table 1). The nucleotide and amino acid similarities between A(H3N2) strains from the 2011 and 2012 seasons and A/Perth/16/2009 were >97.6 and >96.3%, respectively. Meanwhile, the nucleotide and amino acid similarities between the 2013 strains and A/Victoria/361/2011 were 98.7% and 97.7%, respectively. The A(H3N2) strains in 2014 were closely related to A/Texas/50/2012 (98.2% nucleotide and 96.9% amino acid identities).

**Table 1:** Comparison of influenza A(H3N2) nucleotide and amino acid similarities between the vaccine and the circulating Thai strains.

				% identity of HA1	
Year	Clade	No. of strain	Vaccine strain	Nucleotide	Amino acid
2010	1	3	A/Perth/16/2009 (clade 1)	99.2	98.9
2011	3A	1	A/Perth/16/2009	97.9	96.8
(*N* = 24)	3B	6			
	3C.1	17			
2012	3A	1	A/Perth/16/2009	97.6	96.3
(*N* = 16)	3B	2			
	3C.1	6			
	3C.2	7			
2013	3C.2	37	A/Victoria/361/2011 (clade 3C.1)	98.7	97.7
(*N* = 41)	3C.3	4			
2014	3C.2	29	A/Texas/50/2012 (clade 3C.1)	98.2	96.9
(*N* = 36)	3C.3	7			

### Phylogenetic analysis of A(H1N1)pdm09

**Fig 2:**
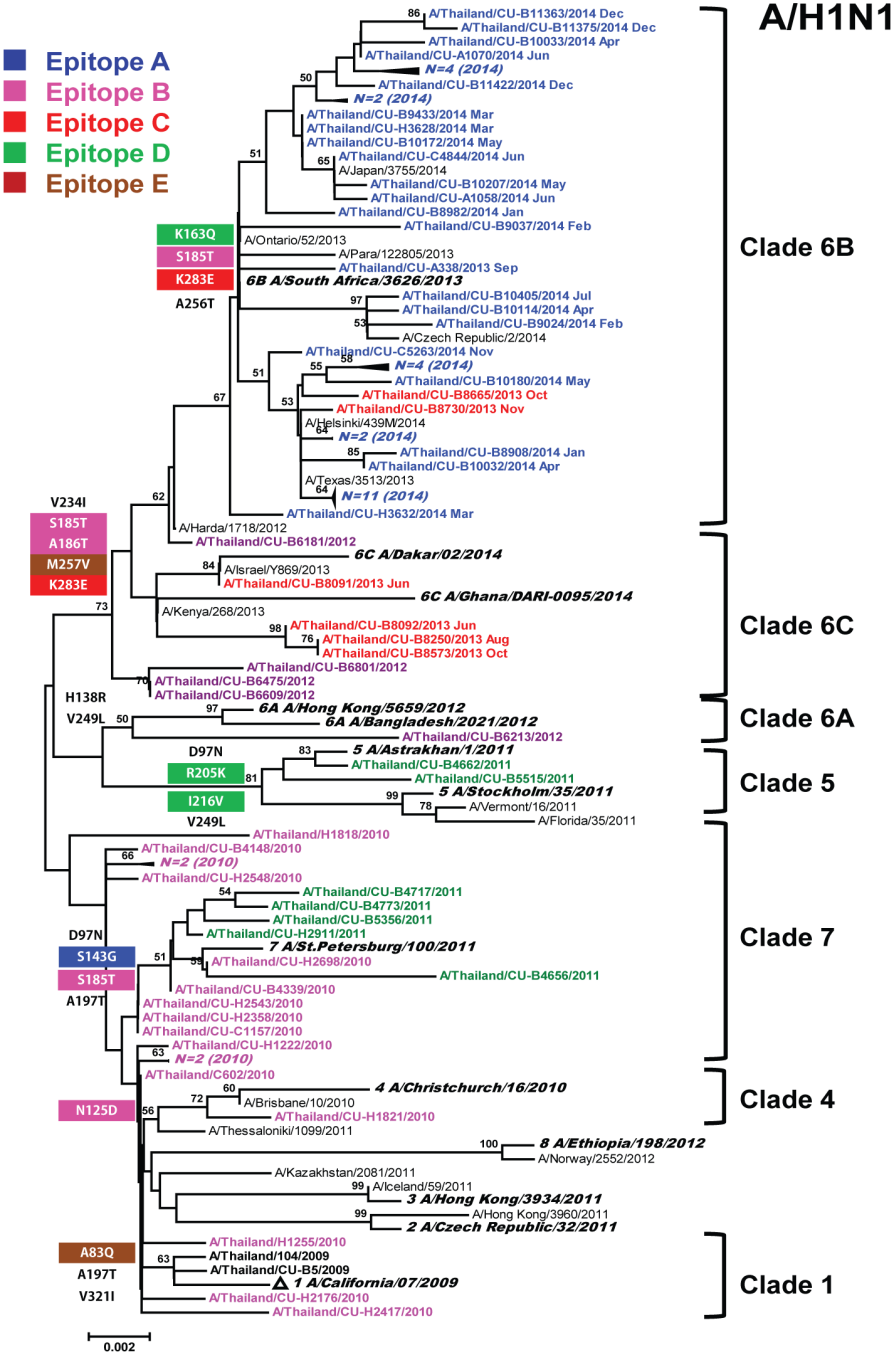
Phylogenetic analysis of the HA1 nucleotide sequences of influenza A(H1N1)pdm09. Sequences from 81 strains isolated in Thailand between 2010 and 2014 (designated A/Thailand/CU) were compared to the reference strains of known clades reported by WHO Influenza Center London (bolded) and the southern hemisphere vaccine strains recommended by WHO (denoted with triangles). Phylogenic tree was generated using maximum likelihood method by HKY+G model. Bootstrap values of 1,000 replicates >50 are indicated at the nodes. Also at the nodes are the signature amino acid changes in different colors according to epitopes. Scale bar represents approximately 0.2% nucleotide difference between close relatives.

To assess the evolution of the A(H1N1)pdm09 during the same period, circulating strains in 2010 (*N* = 18), 2011 (*N* = 7), 2012 (*N* = 5), 2013 (*N* = 7), and 2014 (*N* = 44) seasons were also compared to the vaccine and reference sequences (Fig 2). There were distinct phylogenetic groups of A(H1N1)pdm09 strains between 2010 to 2014. Among the A(H1N1)pdm09 strains, 69% belonged to clade 6 viruses, while 31% grouped into clades 1, 4, 5, and 7. The HA1 sequence of A(H1N1)pdm09 viruses isolated in the 2013–2014 season clustered in genetic clades 6B and 6C. Although both sub-clades were related to the A/California/07/2009 vaccine strain (recommended every year since 2010) and shared > 98.2% nucleotide and > 97.4% amino acid sequence homology, they were slightly different from A/California/07/2009 in that they shared D97N and S185T substitutions (S4 Table). Moreover, sub-clade 6B possessed additional K163Q, K283E, and A256T substitutions, while clade 6C possessed V234I, M257V, and K283E substitutions. No changes were observed in the A(H1N1)pdm09 at residues Y98, T133, W150, H180, and Q223, which are conserved and important in the HA receptor binding pocket of the influenza virus [26].

### Antigenic characterization

**Fig 3:**
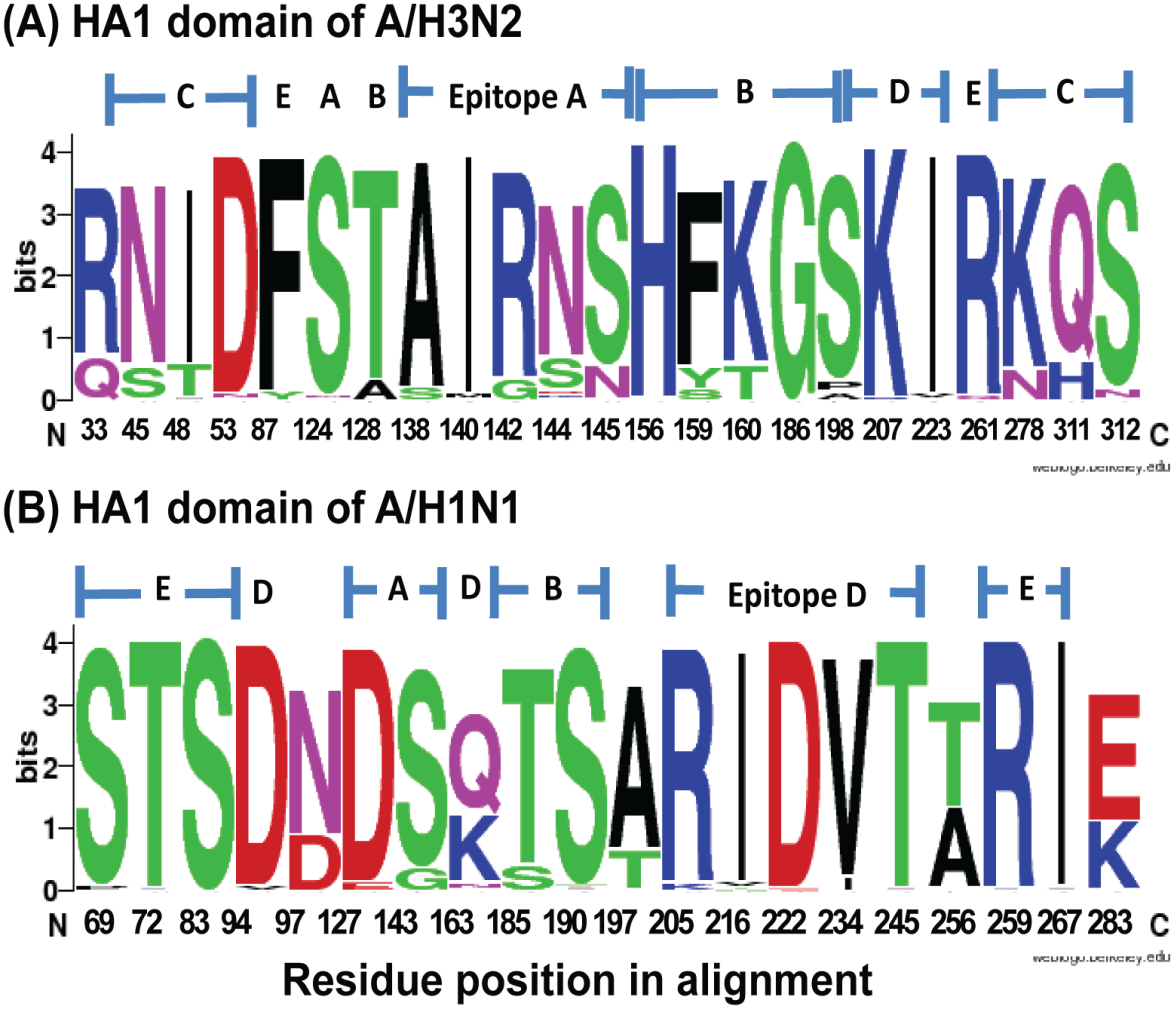
Frequency of amino acid residues found on epitopes A through E in the HA1 protein of influenza virus identified in Thailand during 2010–2014. Residue positions along the *x*-axis for (A) influenza A(H3N2) are based on the A/Perth/16/2009 strain and (B) influenza A(H1N1)pdm09 on the A/California/07/2009. Relative frequency of the amino acid residue at a given position is proportional to the residue height. Residues were colored according to their chemical properties. Polar residues (G, S, T, Y, and C) are green; basic polar residues (K, R, and H) are blue; acidic polar residues (D and E) are red; amide polar residues (Q and N) are purple; and hydrophobic residues (A, V, L, I, P, W, F, and M) are black. Graphics were generated using WebLogo3.

The receptor binding site (RBS) on the HA comprised several highly conserved amino acid residues (Y98, T133, W150, H180, and Q223; numbered according to HA1). Residues at the terminal sialic acid receptor binding sites (RBSs) of all A(H3N2) strains were I226 and S228, while all A(H1N1)pdm09 strains possessed D204 (numbered according to HA0). Additionally, differences in the residues on the A(H3N2) and A(H1N1)pdm09 HA protein were located on the antigenic sites, which comprised epitopes A to E (S3 and S4 Tables). We summarized the relative frequencies of the residues found on the dominant epitope domain on the HA1 of A(H3N2) and A(H1N1)pdm09 (Fig 3). Overall, the A(H3N2) strains displayed more diversity from the accumulated epitope mutations than the A(H1N1)pdm09 strains.

### Selection pressure on A(H3N2) and A(H1N1)pdm09

Assuming that the influenza HA protein is subjected to selection pressure in order to evade the host cell recognition, the rate of change was assessed by the *ω* values in which *ω* < 1 meant that negative or purifying selection was present, *ω* = 1 when selection pressure was neutral, and *ω* >1 when there was positive selection [27]. Analysis showed that the overall *ω* values of the coding HA1 regions of A(H3N2) and A(H1N1)pdm09 were 0.34 and 0.31, respectively. Since the majority of residues in the HA1 domain showed *ω* < 1, this suggested that the amino acids in the HA epitope domain were under purifying selection. Although overall positive selection was not present, specific sites of positive selection were found using SLAC and FEL methods (Table 2). Among the 329 codons in the HA1 domain of the A(H3N2) circulating strains, SLAC showed that only codon 33 (Q/R) was a positively selected site (*ω* = 3.78, *P* = 0.04). FEL identified codons 33 (Q/R), 144 (K/N/D/S), and 198 (A/S) as positively selected sites (*ω* = 8.02 × 10^14^, *P* = 0.02; infinity, *P* = 0.09; and 34.5 × 10^14^, *P* = 0.07; respectively). For A(H1N1)pdm09 strains, only one codon 197 (A/T) was a positively selected site (*ω* = 8.21 × 10^14^, *P* = 0.08) as shown by FEL.

**Table 2:** Positively selected sites on HA1 of influenza A virus among the Thai strains between 2010 and 2014.

	SLAC				FEL			
Subtype	position	d*N*/d*S* ^a^	Normalized d*N*/d*S*	*P*-value^b^	position	d*N*/d*S* ^a^	Normalized d*N*/d*S*	*P*-value^b^
H3N2	33	3.78	11.14	0.04	33	8.02×10^14^	16.3	0.02
					144	Infinity	11	0.09
					198	34.5×10^14^	11.5	0.07
H1N1 pdm 09	N/D	-	-	-	197	8.21×10^14^	46.83	0.08

SLAC, single likelihood ancestor counting; FEL, fixed effects likelihood; N/D, not detected.

^a^ d*N*/d*S* or *ω* is the ratio of synonymous to non-synonymous substitutions.

^b^ *P*-value from the SLAC and FEL results for positive selection level.

### Prediction of glycosylation sites

Ten potential glycosylation sites on the HA1 of A(H3N2) clades 1 and 3A strains were identified at amino acid positions 8, 22, 38, 63, 122, 126, 133, 165, 244, and 285. The K144N substitution occurred in clades 3B and 3C.1 strains, resulting in an increase in the number of glycosylation sites. The S45N substitution appeared in clades 3C.1 and 3C.2 strains, contributing to an increase in the number of glycosylation sites. Loss of a glycosylation site occurred in clade 3C.3 strains due to T128A mutation. The majority of A(H1N1)pdm09 strains possessed 6 potential glycosylation sites in the HA1 domain at amino acid positions 10, 11, 23, 87, 276, and 287, which were present in all but clade 6A. The mutation at N11S among clade 6A strains resulted in a loss of potential glycosylation site at this position. We did not find the D222G mutation commonly associated with increased virulence.

### Estimation of vaccine efficacy for A(H3N2) and A(H1N1)pdm09

To assess the effect of the accumulated mutations on the HA1 domain on the vaccine efficacy in a given year, the *p*_epitope_ method was used to evaluate how closely the vaccine strain resembled the circulating strain (Table 3). Theoretically, when *p*_epitope_ in the dominant epitope is higher than 0.19, the vaccine efficacy becomes negative [10]. For 2010, the *p*_epitope_ between A(H3N2) strains and the A/Perth/16/2009 vaccine strain was 0.045 (epitope E; mutation 261), which suggested a worst-case vaccine efficacy against these strains of 75.96% (*E* = 35.7% of 47%, *p*_epitope_ = 0). For the 2011 and 2012 seasons, HA1 sequences showed antigenic drifts mainly on epitopes A and C (S1 Fig). The *p*_epitope_ of 0.148 (dominant epitope = C substitutions 45, 48, 278, and 312) suggested a worst-case vaccine efficacy against these strains of 22.1% (*E* = 10.4% of 47%, *p*_epitope_ = 0) of that of a perfect match with the A/Perth/16/2009 vaccine strain. Consequently, vaccine efficacy declined by more than half. For 2013, *p*_epitope_ of 0.095 from 30 strains (dominant epitope = B mutations 156 and 186) suggested a worst-case vaccine efficacy against these strains of 49.9% (*E* = 23.4% of 47%, *p*_epitope_ = 0) of that of a perfect match with the A/Victoria/361/2011 vaccine strain. For 2014, the HA1 sequences mostly had a dominant mutation in epitope B (128, 159, 186, and 198) and the *p*_epitope_ of 0.191 with respect to A/Texas/50/2012 vaccine strain, suggesting that the latter poorly matched the multiple circulating strains present that year. The 2015 vaccine strain A/Switzerland/9715293/2013, which belongs to clade 3C.3, was also examined for its ability to match the A(H3N2) strains of the same clade circulating in 2014. The resulting *p*_epitope_ value of 0.052 gave an estimated worst-case vaccine efficacy against strains of 72.3%. However, clade 3C.2 strains of A(H3N2) in 2014 and A/Switzerland/9715293/2013 showed a value of > 0.19, indicating a negative vaccine efficacy against these strains.

**Table 3:** Efficacy among the vaccine strains and number of mutations found on the dominant epitope of influenza A(H3N2) circulating in Thailand.

Year	Vaccine strain	No. of strain	Dominant epitope	No. of mutation	*p*_epitope_	Efficacy	Vaccine efficacy (47%)	Vaccine efficacy (100%)
2010	A/Perth/16/2009	2	E	1	0.0455	0.3577	35.77	75.96
(*N* = 3)		1	A	1	0.0526	0.3400	34.00	72.34
2011	A/Perth/16/2009	6	A	2	0.1053	0.2100	21.00	44.68
(*N* = 24)		14	C	4	0.1481	0.1041	10.41	22.14
		4	A	3	0.1579	0.0800	8.00	17.02
2012	A/Perth/16/2009	3	A	2	0.1053	0.2100	21.00	44.68
(*N* = 16)		11	C	4	0.1481	0.1041	10.41	22.14
		2	A	3	0.1579	0.0800	8.00	17.02
2013	A/Victoria/361/2011	30	B	2	0.0952	0.2348	23.48	49.95
(*N* = 41)		2	A	2	0.1053	0.2100	21.00	44.68
		3	C	3	0.1111	0.1956	19.56	41.61
		4	B	3	0.1429	0.1171	11.71	24.92
		1	A	3	0.1579	0.0800	8.00	17.02
		1	B	4	0.1905	−0.0005	−0.05	−0.10
2014	A/Texas/50/2012	1	B	2	0.0952	0.2348	23.48	49.95
(*N* = 36)		8	B	3	0.1429	0.1171	11.71	24.92
		17	B	4	0.1905	−0.0005	−0.05	−0.10
		9	B	5	0.2381	−0.1181	−11.81	−25.13
		1	A	7	0.3684	−0.4400	−44.00	−93.62
	A/Switzerland/9715293/2013	6	A	1	0.0526	0.3400	34.00	72.34
		1	B	2	0.0952	0.2348	23.48	49.95
		8	A	3	0.1579	0.0800	8.00	17.02
		1	B	4	0.1905	−0.0005	−0.05	−0.10
		14	A	4	0.2105	−0.0500	−5.00	−10.64
		4	B	5	0.2381	−0.1181	−11.81	−25.13
		1	A	5	0.2632	−0.1800	−18.00	−38.30
		1	A	11	0.5263	−0.8300	−83.00	−176.60

In contrast, comparison between the A(H1N1)pdm09 strains circulating between 2010–2014 and A/California/07/2009 vaccine strain yielded the *p*_epitope_ of 0.045. This was attributed to an amino acid substitution at 185 on the dominant epitope B (S2 Fig) and suggested a worst-case vaccine efficacy against these strains of 89.7% (E = 47.6% of 53%, *p*_epitope_ = 0) of that of a perfect match with the vaccine strain (Table 4). In all, 12.3% of the strains obtained between 2010–2014 (10/81) possessed dominant mutation in epitope E at position 83, which gave an estimated worst-case vaccine efficacy against the virus of 93.4% (E = 49.5% of 53%, *p*_epitope_ = 0) of a perfect match with the vaccine strain. In summary, HA1 sequences of A(H1N1)pdm09 from recent years showed antigenic changes mainly on epitope B and one or two amino acid mutations on other epitopes. Taken together, these results suggested that the past and current vaccine provided optimal protection against A(H1N1)pdm09 strains that circulated in Thailand.

**Table 4:** Vaccine efficacy and number of mutations in dominant epitope of influenza A(H1N1)pdm09 circulating in Thailand compared with A/California/07/2009 vaccine strain.

Year	No. of strain	Dominant epitope	No. of mutations	*p*_epitope_	Efficacy	Vaccine efficacy (53%)	Vaccine efficacy (100%)
2010	11	B	1	0.0455	0.4759	47.59	89.79
(*N* = 18)	1	C	2	0.0606	0.4579	45.79	86.39
	6	E	1	0.0294	0.4950	49.50	93.40
2011	2	A	2	0.0833	0.4308	43.08	81.29
(*N* = 7)	3	B	1	0.0455	0.4759	47.59	89.79
	1	B	2	0.0909	0.4218	42.18	79.59
	1	E	1	0.0294	0.4950	49.50	93.40
2012	5	B	1	0.0455	0.4759	47.59	89.79
2013	3	B	1	0.0455	0.4759	47.59	89.79
(*N* = 7)	1	C	2	0.0606	0.4579	45.79	86.39
	3	E	2	0.0588	0.4600	46.00	86.79
2014	44	B	1	0.0455	0.4759	47.59	89.79

## Discussion

Since its emergence in 1968, influenza A(H3N2) strain has been the predominant circulating influenza subtype between 2011 and 2014 [28]. In contrast, the A(H1N1) subtype was first documented in Thailand in May 2009 and continued to circulate until 2010. Although the (H1N1)pdm09-like A/California/7/2009 has remained the recommended vaccine strain for the past several years, we identified changes on the HA1 of A(H1N1)pdm09 Thai strains belonging to epitopes B (S185T) and E (P83S). Additional observed changes at residues 97 (D97N) and 197 (A197T) previously implicated in the adaptive mutation or immune escape continued to persist (71% and 18%, respectively) [29]. In this study, the vaccine efficacy for A(H1N1)pdm09 strains of 79.6–93.4% was higher than that for the A(H3N2). This is concordant with previous studies, which estimated the vaccine efficacy using serologically based methods and suggested a moderate to high vaccine effectiveness for influenza A(H1N1)pdm09 during the 2010–2014 seasons [30, 31].

We found that influenza clusters on the phylogenetic tree is mostly chronological. The influenza A(H3N2) strains found in Thailand belonging to at least two phylogenetic sub-clades (1 and 3), which co-circulated between 2011 and 2014. During the 2010 season, only strains in clade 1 were identified. In 2011–2012, A(H3N2) sub-clade 3C strains appeared. Accumulated amino acid variations on epitope C in the HA1 domain allowed it to drift away from the A/Perth/16/2009-like strain (the vaccine strain of 2010, 2011, and 2012 seasons). As a result, the vaccine strain was changed to the A/Victoria/361/2011-like strain for 2013 season. However, all A(H3N2) strains circulated in 2013 and 2014 seasons belonged to the new emerging sub-clades 3C.2 (A/Hong Kong/146/2013-like strain) and 3C.3 (A/Samara/73/2013-like strain), which were different from the sub-clade 3C.1 A/Victoria/361/2011 and A/Texas/50/2012 strains used for the vaccines in those years. This was consistent with the *p*_epitope_ >0.19 obtained from the analysis, which revealed high antigenic drift and subsequently resulted in negative vaccine efficacy.

Genetic evolution of influenza virus appears gradual, but antigenic changes were found to occur more abruptly [32]. For example, one single amino acid substitution in the case of N145K on the HA1 of A(H3N2) can characterize the difference between clades. It is also known that the antigenic variation of H3 occurs more frequently than H1. The average amino acid substitution rate of the HA protein is 3.6 per year for A(H3N2) and 2.45 per year for A(H1N1) [32–33]. One reason may be that more individuals are susceptible to the relatively novel A(H1N1)pdm09 strain, and therefore the weaker immnue pressure has resulted in the slow rate of the viral evolution.

New influenza variants are thought to drift considerably from the parental strain when they displayed four or more amino acid mutations on at least two epitope domains on the HA1 protein [34]. Alternatively, antigenic drift variants can result from a change in the antigenic site in combination with a mutation in the RBS, which interacts with the sialic acid on the cell surface [35]. We found that the HA1 sequences from the A(H3N2) strains during the 2011–2012 season possessed seven amino acid changes on four epitopes including epitope C (S45N, T48I, N278K, and N312S), epitope B (A198S), epitope D (V223I), and Q33R. In the 2013–2014 seasons, antigenic drift also occurred due to at least four amino acid mutations on epitope B combined with additional mutations on epitope A (R142G), epitope B (T128A), and epitope C (N278K). It is noteworthy that T128A had previously been observed in Fujian strain, which was associated with high mortality rate in children [36].

Further analysis of the HA1 from A(H3N2) strains revealed three positively selected codons (33, 144, and 198), suggesting that these sites were immune-escaped mutants. The N144D substitution was not unique to the strains found in Thailand as it was observed among isolates in Europe and Africa during 2010–2011 [37]. The resulting glycosylation at position 144 was previously implicated in the antigenic change in A/Fujian/411/02-like strains from the 2002–2003 seasons [38]. Compared to A/Perth/16/2009, two amino acid mutations involving A128T and N45S could effectively alter the glycosylation pattern, providing evolutionary advantage to the virus including more effective masking of viral epitopes, stabilization of polymeric HA structures, regulation of the receptor binding domain, and balancing the binding activity of HA with the release activity of neuraminidase [39]. This was evident when it was observed that the loss of the glycosylation site at 128 of HA1 was associated with loss of antibody recognition [40].

The vaccine efficacy between A/Perth/16/2009 vaccine strain and A(H3N2) strains circulated in Thailand in 2010 of 75.96% is consistent with a moderate vaccine efficacy reported for the trivalent inactivated influenza vaccines in 2010–2011 in Thailand [41] and a moderate protection against subtype-specific A/H3 reported in the U.S. [42]. During the 2011–2012 seasons, the dominant epitope change from A to C relative to the A/Perth/16/2009 subsequently resulted in a decline in the percentage of perfect-match vaccine efficacy (44.6% and 22.1%, respectively). Furthermore, the antigenic sites of the circulating A(H3N2) strains in 2013 and 2014 drifted from epitope C to B compared to the vaccine strains. In 2013, the reference vaccine strain had to be changed from A/Perth/16/2009 to A/Victoria/361/2011, which appeared to moderately improve the perfect-match vaccine efficacy (49.9%). This was consistent with the results from an epidemiological cohort study showing vaccine efficacy in the 2011–2012 (55%) and 2012–2013 (64%) seasons among Thai children [9]. Meanwhile, the vaccine strain chosen in 2014 (A/Texas/50/2012-like) did not improve the perfect-match vaccine efficacy, which was fairly low (approximately 24.9%). Moreover, the *p*_epitope_ values for the majority of the HA1 sequences in 2014 (75%) was > 0.19 [10] and, therefore, the vaccine efficacy became negative. This has occurred in the past whereby the outbreak of the Sydney/5/79 strain yielded the value of *p*_epitope_ of 0.238; hence, the vaccine efficacy was −17% compared with the 1997–1998 northern hemisphere influenza vaccine [43]. Taken together, the emergence of multiple circulating strains in 2014 contributed to the reduced vaccine efficacy in Thailand that year and was reflected in the weekly morbidity and mortality report from the U. S. Centers for Disease Control and Prevention, which suggested that the 2014–15 influenza vaccine strain A/Texas/50/2012 was essentially ineffective against the circulating A(H3N2) strains [44].

There are several limitations in this study. Since there is no general consensus on the epitope regions for A(H1N1)pdm09, we estimated the antigenic drift and vaccine efficacy based on the mutation of the dominant epitope by mapping epitopes A-E from H3 onto the pandemic A/California/04/2009 strain. Our results therefore require validation using alternative models with differently defined epitope regions [45–46]. The greater number of A(H3N2) strains analyzed in this study may have contributed to more mutations observed on the epitope domains of the A(H3N2) than the A(H1N1). Finally, the assessment of vaccine efficacy relied on the comparison of the circulating influenza strains to the vaccine strains chosen annually, therefore this measurement is not absolute as antigenic diversity have not always been predictive of the vaccine effectiveness. In conclusion, continued influenza surveillance, molecular evolution analysis, and antigenic distance measurement of the dominant influenza A strains in circulation will help refine the interpretation of vaccine efficacy and improve the yearly influenza vaccine.

## Supporting Information

S1 Fig Positions of mutations in the dominant epitope of HA1 influenza A(H3N2) compared with vaccine strains. (PDF)

S2 Fig Positions of mutations in the dominant epitope of HA1 influenza A(H1N1)pdm09 compared with A/California/07/2009 vaccine strain. (PDF)

S1 Table Primers used for conventional PCR amplification of the HA gene of influenza A(H3N2) and A(H1N1)pdm09 strains circulating in Thailand. (DOCX)

S2 Table Accession numbers in GenBank and GISAID of HA influenza A(H3N2) and A(H1N1)pdm09 gene sequences used for phylogenetic analysis. (DOCX)

S3 Table Amino acid changes observed in the antigenic sites (epitopes A through E) of the HA protein of 120 influenza A(H3N2) strains. (DOCX)

S4 Table Amino acid changes observed in the antigenic sites (epitopes A through E) of the HA protein of 81 influenza A(H1N1)pdm09 strains. (DOCX)
