# Supplementary material for: Assessing Antigenic Drift of Seasonal Influenza A(H3N2) and A(H1N1)pdm09 Viruses
Source: PLoS One. 2015 Oct 6;10(10):e0139958. doi: 10.1371/journal.pone.0139958 (PMC4594909; doi:10.1371/journal.pone.0139958)
Supplement: S1 Fig — (PDF) [file pone.0139958.s001.pdf]

S1 Fig. Positions of mutations in the dominant epitope of HA1 influenza A(H3N2) compared with vaccine strains.

Year	Vaccine strain	No of strain	Dominant Epitope	Differing Residues
2010	A/Perth/16/2009	2	E	261
(N=3)		1	A	124
2011	A/Perth/16/2009	6	A	144 145
(N=24)		1	A	124 144 145
		3	A	140 144 145
		14	C	45 48 278 312
2012	A/Perth/16/2009	3	A	144 145
(N=16)		1	A	124 144 145
		1	A	140 144 145
		11	C	45 48 278 312
2013	A/Victoria/361/2011	1	A	143 145
(N=41)		1	A	144 145
		1	A	124 142 145
		30	B	156 186
		1	B	156 160 186
		1	B	156 186 187
		2	B	128 156 186
		1	B	128 156 157 186
		2	C	45 48 278
		1	C	278 297 304
2014	A/Texas/50/2012	1	A	126 133 144 145 146 150 152
(N=36)		1	B	128 186
		8	B	128 186 198
		5	B	128 159 160 186
		12	B	128 159 186 198
		1	B	128 156 159 186 198
		8	B	128 159 160 186 198
2014	A/Switzerland/9715293/2013	6	A	140
(N=36)		1	B	156 186
		8	A	138 140 142
		14	A	138 140 142 144
		1	B	128 159 186 198
		1	A	135 138 140 142 144
		4	B	128 159 160 186 198
		1	A	10 126 133 138 140 142 144 145 146 150 152
