# Supplementary material for: Assessing Antigenic Drift of Seasonal Influenza A(H3N2) and A(H1N1)pdm09 Viruses
Source: PLoS One. 2015 Oct 6;10(10):e0139958. doi: 10.1371/journal.pone.0139958 (PMC4594909; doi:10.1371/journal.pone.0139958)
Supplement: S2 Fig — (PDF) [file pone.0139958.s002.pdf]

S2 Fig. Positions of mutations in the dominant epitope of HA1 influenza A(H1N1)pdm09 compared with A/California/07/2009 vaccine strain

Year	No. of strain	Dominant Epitope	No. of mutation	Differing Residues	
2010	9	B	1	185	
(N=18)	2	B	1	190	
	1	C	2	38	277
	6	E	1	83	
2011	2	A	2	127	143
(N=7)	3	B	1	185	
	1	B	2	183	185
	1	E	1	83	
2012	5	B	1	185	
2013	3	B	1	185	
(N=7)	1	C	2	283	302
	2	E	2	83	69
	1	E	2	83	263
2014	44	B	1	185	
