# Supplementary material for: Assessing Antigenic Drift of Seasonal Influenza A(H3N2) and A(H1N1)pdm09 Viruses
Source: PLoS One. 2015 Oct 6;10(10):e0139958. doi: 10.1371/journal.pone.0139958 (PMC4594909; doi:10.1371/journal.pone.0139958)
Supplement: S1 Table — (DOCX) [file pone.0139958.s003.docx]

**S1 Table: Primers used for conventional PCR amplification of the HA gene of influenza A(H3N2) and A(H1N1)pdm09 strains circulating in Thailand.**

| **Primer Name** | **Nucleotide Sequences (5’🡪3’)** | **PCR product (bp)** |
| --- | --- | --- |
| HA_F5’  H3_R797 | AGCAAAAGCAGGGGAAAATAAAAGCA  TCCCGGATTTACTATTGTCCA | 797 |
| H3_F598  H3_R1164 | TTGACAAATTGTACATTTGGGG  GCTTTTGAGATCTGCTGCTTG | 566 |
| H3_F1013  HA_R3’ | CACTCTGAAATTGGCAACAGG  AGTAGAAACAAGGGTGTTTTTAACTAC | 688 |
| HA_F1  H1_R694 | AGCAAAAGCAGGGGAAAATAAAAGCA  TCTTGATGACCCCACAAAAACATA | 694 |
| H1_F510  H1_R1126 | AGCTTCTACARAAATTTAATATGGCT  CATCCATCTACCATCCCTGTCCA | 616 |
| H1_F923  H1_R1548 | AACACCCAAGGGTGCTATAAACA  ATTTTGGGTAGTCATAAGTCCCATT | 625 |
| H1_F1398  H1-R1780 | GATTCAAATGTGAAGAACTTATATGA  AGTAGAAACAAAGGGTGTTTTTTCTCATGT | 382 |
