# Supplementary material for: Assessing Antigenic Drift of Seasonal Influenza A(H3N2) and A(H1N1)pdm09 Viruses
Source: PLoS One. 2015 Oct 6;10(10):e0139958. doi: 10.1371/journal.pone.0139958 (PMC4594909; doi:10.1371/journal.pone.0139958)
Supplement: S2 Table — (DOCX) [file pone.0139958.s004.docx]

**S2 Table: Accession numbers in GenBank and GISAID of HA influenza A(H3N2) and A(H1N1)pdm09 gene sequences used for phylogenetic trees analysis.**

| **Name** | **Accession No.** |
| --- | --- |
| A/H3N2-TH strains (2010) | CY074950 CY074958 CY074966 |
| A/H1N1-TH strains (2010) | CY080307 CY080299 CY089429 CY089437  CY081156 CY074982 CY080323 CY080339  CY088801 CY088809 CY088816 CY088823  CY088830 CY088838 CY089447 CY089455  CY089463 CY080315 CY080331 |
| A/H3N2 reference and vaccine strains | KC892248 KC535440 KC882860 KC892582 GQ293081 EU021268 EU021276 EU625364  EPI318272 EPI346607 EPI353906 EPI467994 EPI426061 EPI539576 EPI460558 EPI540526 EPI539874 EPI319276 EPI326115 EPI232453 EPI367105 EPI160218 EPI165489 GQ902809 |
| A/H1N1 reference and vaccine strains | EPI319447 EPI326206 EPI280344 EPI319590  EPI382424 EPI466626 EPI539470 EPI541029  EPI316435 EPI319527 EPI450810 EPI466545  EPI331059 EPI239666 EPI253705 EPI279895  FJ969540 GQ205436 GQ166661 |
