# Supplementary material for: Assessing Antigenic Drift of Seasonal Influenza A(H3N2) and A(H1N1)pdm09 Viruses
Source: PLoS One. 2015 Oct 6;10(10):e0139958. doi: 10.1371/journal.pone.0139958 (PMC4594909; doi:10.1371/journal.pone.0139958)
Supplement: S3 Table — (DOCX) [file pone.0139958.s005.docx]

**S3 Table: Amino acid changes observed in the antigenic sites (epitopes A through E) of the HA protein of 120 influenza A(H3N2) strains.**

| **Amino acid position** | **33** | **45** | **48** | **53** | **87** | **124** | **128** | **138** | **140** | **142** | **144** | **145** | **156** | **159** | **160** | **186** | **198** | **207** | **223** | **261** | **278** | **311** | **312** |
| --- | --- | --- | --- | --- | --- | --- | --- | --- | --- | --- | --- | --- | --- | --- | --- | --- | --- | --- | --- | --- | --- | --- | --- |
| **Antigenic sites** |  | **C** | **C** | **C** | **E** | **A** | **B** | **A** | **A** | **A** | **A** | **A** | **B** | **B** | **B** | **B** | **B** | **D** | **D** | **E** | **C** | **C** | **C** |
| **A/Perth/16/2009** | **Q** | **S** | **T** | **D** | **F** | **S** | **T** | **A** | **I** | **R** | **K** | **N** | **H** | **F** | **K** | **G** | **A** | **K** | **V** | **R** | **N** | **Q** | **N** |
| A/Victoria/361/2011 | . | N | I | . | . | . | . | . | . | . | N | . | Q | . | . | V | S | . | I | . | . | . | S |
| A/Texas/50/2012 | R | N | I | . | . | . | N | . | . | . | N | . | . | . | . | V | P | . | I | . | K | . | S |
| A/Thailand/CU-H1285/2010 | . | . | . | . | . | . | . | . | . | . | . | . | . | . | . | . | . | . | . | Q | . | . | . |
| A/Thailand/CU-H1443/2010 | . | . | . | . | . | N | . | . | . | . | . | . | . | . | . | . | . | . | . | Q | . | . | . |
| A/Thailand/CU-H1817/2010 | . | . | . | . | . | . | . | . | . | . | . | . | . | . | . | . | . | . | . | Q | . | . | . |
| A/Thailand/CU-B4730/2011 | . | . | . | . | . | . | . | . | . | . | N | S | . | . | . | . | S | . | I | . | . | . | S |
| A/Thailand/CU-H2973/2011 | . | . | . | . | . | . | . | . | . | . | N | S | . | . | . | . | S | . | I | . | . | . | S |
| A/Thailand/CU-B4844/2011 | R | N | I | . | . | . | . | . | . | . | N | . | . | . | . | . | S | . | I | . | K | . | S |
| A/Thailand/CU-H2989/2011 | R | N | I | . | . | . | . | . | . | . | N | . | . | . | . | . | S | . | I | . | K | . | S |
| A/Thailand/CU-B4836/2011 | . | . | . | . | . | N | . | . | . | . | N | S | . | . | . | . | S | . | I | . | . | . | S |
| A/Thailand/CU-B4929/2011 | R | N | I | . | . | . | . | . | . | . | N | . | . | . | . | . | S | . | I | . | K | . | S |
| A/Thailand/CU-B5021/2011 | . | N | I | . | . | . | . | . | M | . | N | S | . | . | . | . | S | . | I | . | . | . | S |
| A/Thailand/CU-B5105/2011 | . | . | . | . | . | . | . | . | . | . | N | S | . | . | . | . | S | . | I | . | . | . | S |
| A/Thailand/CU-H3020/2011 | . | . | . | . | . | . | . | . | . | . | N | S | . | . | . | . | S | . | I | . | . | . | S |
| A/Thailand/CU-B5436/2011 | . | . | . | . | . | . | . | . | . | . | N | S | . | . | . | . | S | . | I | . | K | . | S |
| A/Thailand/CU-B5465/2011 | R | N | I | . | . | . | . | . | . | . | N | . | . | . | . | . | S | . | I | . | K | . | S |
| A/Thailand/CU-B5541/2011 | R | N | I | . | . | . | . | . | . | . | N | . | . | . | . | . | S | . | I | . | K | . | S |
| A/Thailand/CU-B5593/2011 | R | N | I | . | . | . | . | . | . | . | N | . | . | . | . | . | S | . | I | . | K | . | S |
| A/Thailand/CU-B5614/2011 | R | N | I | . | . | . | . | . | . | . | N | . | . | . | . | . | S | . | I | . | K | . | S |
| A/Thailand/CU-B5697/2011 | R | N | I | . | . | . | . | . | . | . | N | . | . | . | . | . | S | . | I | . | K | . | S |
| A/Thailand/CU-B5735/2011 | . | N | M | . | . | . | . | . | M | . | N | S | . | . | . | . | S | . | I | . | . | . | S |
| A/Thailand/CU-B5773/2011 | . | . | . | . | . | . | . | . | . | . | D | S | . | . | . | . | . | . | I | . | . | . | . |
| A/Thailand/CU-B5848/2011 | R | N | I | . | . | . | . | . | . | . | N | . | . | . | . | . | S | . | I | . | K | . | S |
| A/Thailand/CU-B5873/2011 | R | N | I | . | . | . | . | . | . | . | N | . | . | . | . | . | S | . | I | . | K | . | S |
| A/Thailand/CU-B5898/2011 | R | N | I | . | . | . | . | . | . | . | N | . | . | . | . | . | S | . | I | . | K | . | G |
| A/Thailand/CU-B5900/2011 | R | N | I | . | . | . | . | . | . | . | N | . | . | . | . | . | S | . | I | . | K | . | S |
| A/Thailand/CU-B5909/2011 | R | N | I | . | . | . | . | . | . | . | N | . | . | . | . | . | S | . | I | . | K | . | S |
| A/Thailand/CU-B5928/2011 | R | N | I | . | . | . | . | . | . | . | N | . | . | . | . | . | S | . | I | . | K | . | S |
| A/Thailand/CU-C2417/2011 | . | N | I | . | . | . | . | . | M | . | N | S | . | . | . | . | S | . | I | . | . | . | S |
| A/Thailand/CU-H3141/2012 | . | . | . | . | . | . | . | . | . | . | D | S | . | . | . | . | . | . | I | . | . | . | . |
| A/Thailand/CU-B6091/2012 | R | N | I | . | . | . | . | . | . | . | N | . | . | . | . | . | S | . | I | . | K | . | S |
| A/Thailand/CU-B6251/2012 | R | N | I | . | . | . | . | . | . | . | N | S | . | . | . | . | S | . | I | . | K | . | S |
| A/Thailand/CU-B6274/2012 | R | N | I | . | . | . | . | . | . | . | N | . | . | . | . | . | S | . | I | . | K | . | S |
| A/Thailand/CU-B6309/2012 | . | . | . | N | . | . | . | . | . | . | N | S | . | . | . | . | S | . | I | . | . | . | S |
| A/Thailand/CU-B6780/2012 | . | . | . | . | . | . | . | . | . | . | N | S | . | . | . | . | S | . | I | . | . | . | S |
| A/Thailand/CU-H3368/2012 | R | N | I | . | . | . | . | . | T | . | N | S | . | . | . | . | S | . | I | . | K | . | S |
| A/Thailand/CU-B6936/2012 | R | N | I | . | . | . | . | . | . | . | N | S | . | . | . | . | S | . | I | . | K | . | S |
| A/Thailand/CU-H3434/2012 | R | N | I | . | Y | . | . | . | . | . | N | S | . | . | . | . | S | . | I | . | K | . | S |
| A/Thailand/CU-H3435/2012 | R | N | I | . | . | . | . | . | . | G | N | S | . | . | . | . | S | . | I | . | K | . | S |
| A/Thailand/CU-H3453/2012 | R | N | I | . | . | . | . | . | . | . | N | S | . | . | . | . | S | . | I | . | K | . | S |
| A/Thailand/CU-B7189/2012 | R | N | I | . | Y | . | . | . | . | . | N | S | . | . | . | . | S | . | I | . | K | . | S |
| A/Thailand/CU-H3490/2012 | R | N | I | . | Y | . | . | . | . | . | N | S | . | . | . | . | S | . | I | . | K | . | S |
| A/Thailand/CU-B7235/2012 | R | N | I | . | Y | . | . | . | . | . | N | S | . | . | . | . | S | . | I | . | K | . | S |
| A/Thailand/CU-B7269/2012 | R | N | I | . | Y | . | . | . | . | . | N | S | . | . | . | . | S | . | I | . | K | . | S |
| A/Thailand/CU-B7367/2012 | R | N | I | . | . | . | . | . | . | . | N | S | . | . | . | . | S | . | I | . | K | . | S |
| A/Thailand/CU-B7418/2013 | R | N | I | . | . | . | . | . | . | . | N | S | . | . | . | . | S | . | I | . | K | . | S |
| A/Thailand/CU-B7483/2013 | R | N | I | . | . | . | . | . | . | . | N | S | . | . | . | . | S | . | I | . | K | . | S |
| A/Thailand/CU-B7536/2013 | R | N | I | . | . | . | . | . | . | . | N | S | . | . | . | . | S | . | I | . | K | . | S |
| A/Thailand/CU-B7585/2013 | R | N | I | . | . | . | . | . | . | . | N | S | . | . | . | . | S | . | I | . | K | . | S |
| A/Thailand/CU-B7596/2013 | R | N | I | . | . | . | . | . | . | . | N | S | . | . | . | . | S | . | I | . | K | . | S |
| A/Thailand/CU-B7646/2013 | . | N | I | . | . | . | . | . | . | . | N | S | . | . | . | . | S | . | I | . | K | . | S |
| A/Thailand/CU-B7755/2013 | R | N | I | . | . | . | . | . | . | . | N | S | . | . | . | . | S | . | I | . | K | . | S |
| A/Thailand/CU-B7765/2013 | R | N | I | . | . | . | . | . | . | . | N | S | . | . | . | . | S | . | I | . | K | . | S |
| A/Thailand/CU-B7853/2013 | R | N | I | . | . | . | A | . | . | G | N | S | . | . | . | . | S | . | I | . | K | . | S |
| A/Thailand/CU-B7885/2013 | R | N | I | . | . | . | . | . | . | . | N | S | . | . | . | . | S | . | I | . | K | . | S |
| A/Thailand/CU-B7937/2013 | R | N | I | . | . | . | A | . | . | G | N | S | . | . | . | . | S | . | I | . | K | . | S |
| A/Thailand/CU-A7/2013 | . | . | . | . | . | . | . | . | . | . | N | S | . | . | . | . | S | . | I | . | K | . | S |
| A/Thailand/CU-A24/2013 | R | N | I | . | . | . | . | . | . | . | N | S | . | . | . | . | S | . | I | . | K | . | S |
| A/Thailand/CU-A114/2013 | R | N | I | . | . | . | . | . | . | . | N | S | . | . | . | . | S | . | I | . | K | . | S |
| A/Thailand/CU-B7992/2013 | . | . | . | . | . | . | . | . | . | . | N | S | . | . | . | . | S | . | L | . | . | . | S |
| A/Thailand/CU-A134/2013 | R | N | I | . | . | . | . | . | . | . | N | S | . | . | . | . | S | . | I | . | K | . | S |
| A/Thailand/CU-A153/2013 | R | N | I | . | . | . | . | . | . | . | N | S | . | . | . | . | S | . | I | . | K | . | S |
| A/Thailand/CU-A164/2013 | R | N | I | . | . | . | . | . | . | . | N | S | . | . | . | . | S | R | I | . | K | . | S |
| A/Thailand/CU-A166/2013 | R | N | I | . | . | . | . | . | . | . | N | S | . | . | . | . | S | . | I | . | K | . | S |
| A/Thailand/CU-A182/2013 | R | N | I | . | . | . | . | . | . | . | N | S | . | . | . | . | S | . | I | . | K | . | S |
| A/Thailand/CU-A196/2013 | . | . | . | . | . | . | . | . | . | . | N | S | . | . | . | . | S | R | I | . | K | . | S |
| A/Thailand/CU-B8121/2013 | R | N | I | . | . | . | . | . | . | . | N | S | . | . | . | . | S | . | I | . | K | . | S |
| A/Thailand/CU-B8127/2013 | . | . | . | . | . | . | . | . | . | . | N | S | . | . | . | . | S | . | I | . | . | . | S |
| A/Thailand/CU-B8222/2013 | R | N | I | . | . | . | . | . | . | . | N | S | . | . | . | . | S | . | I | . | K | . | S |
| A/Thailand/CU-A305/2013 | R | N | I | . | . | . | . | . | . | . | N | S | . | . | . | . | S | . | I | . | K | . | S |
| A/Thailand/CU-C4087/2013 | R | N | I | . | . | . | . | . | . | . | D | S | . | . | . | . | S | . | I | . | K | . | S |
| A/Thailand/CU-B8235/2013 | R | N | I | . | . | G | A | . | . | G | N | S | . | . | . | . | S | . | I | . | K | . | S |
| A/Thailand/CU-B8236/2013 | R | N | I | . | . | . | . | . | . | . | N | S | . | . | . | . | S | . | I | . | K | . | S |
| A/Thailand/CU-B8364/2013 | R | N | I | . | . | . | . | . | . | . | N | S | . | . | . | . | S | . | I | . | K | . | S |
| A/Thailand/CU-B8518/2013 | R | N | I | . | . | . | . | . | . | . | N | S | . | . | . | . | S | . | I | . | K | . | S |
| A/Thailand/CU-B8525/2013 | R | N | I | . | . | . | . | . | . | . | N | S | . | . | . | . | S | . | I | . | K | . | S |
| A/Thailand/CU-A411/2013 | R | N | I | . | . | . | . | . | . | . | N | S | . | . | . | . | S | . | I | . | K | . | S |
| A/Thailand/CU-A459/2013 | R | N | I | N | . | . | . | . | . | . | N | S | . | . | . | . | S | . | I | . | K | . | S |
| A/Thailand/CU-H3567/2013 | R | N | I | . | . | . | . | . | . | . | N | S | . | . | . | . | S | . | I | . | K | . | S |
| A/Thailand/CU-H3574/2013 | R | N | I | . | . | . | . | . | . | . | N | S | . | . | . | . | S | . | I | . | K | . | S |
| A/Thailand/CU-B8736/2013 | R | N | I | . | . | . | A | . | . | G | N | S | . | . | . | . | S | . | I | . | K | . | S |
| A/Thailand/CU-B8745/2013 | R | N | I | . | . | . | . | . | . | . | N | S | . | . | . | . | S | . | I | . | K | . | S |
| A/Thailand/CU-B8772/2013 | . | N | I | . | . | . | . | . | . | . | N | S | . | . | . | . | S | . | I | . | K | . | S |
| A/Thailand/CU-C4364/2013 | R | N | I | . | . | . | . | . | . | . | N | S | . | . | . | . | S | . | I | . | K | . | S |
| A/Thailand/CU-H3580/2013 | R | N | I | . | . | . | . | . | . | . | S | S | . | . | T | . | S | . | I | . | K | H | S |
| A/Thailand/CU-H3584/2013 | R | N | I | G | . | . | . | . | . | . | N | S | . | . | . | . | S | . | I | . | K | . | S |
| A/Thailand/CU-A598/2014 | R | N | I | . | . | . | . | . | . | . | N | S | . | . | . | . | S | . | I | . | K | . | S |
| A/Thailand/CU-B8849/2014 | R | N | I | . | . | . | . | . | . | . | N | S | . | . | . | . | S | . | I | . | K | . | S |
| A/Thailand/CU-C4406/2014 | R | N | I | . | . | . | . | . | . | . | N | S | . | . | . | . | S | . | I | . | K | . | S |
| A/Thailand/CU-C4492/2014 | R | N | I | . | . | . | . | . | . | . | N | S | . | . | . | . | S | . | I | . | K | . | S |
| A/Thailand/CU-H3595/2014 | R | N | I | . | . | . | . | . | . | . | N | S | . | . | . | . | S | . | I | . | K | . | S |
| A/Thailand/CU-C4507/2014 | R | N | I | . | . | . | . | . | . | . | N | S | . | . | . | . | S | . | I | . | K | . | S |
| A/Thailand/CU-C4546/2014 | R | N | I | . | . | . | . | . | . | . | N | S | . | . | . | . | S | . | I | . | K | . | S |
| A/Thailand/CU-H3611/2014 | R | D | I | . | . | . | . | . | . | . | S | S | . | . | T | . | S | . | I | . | K | H | S |
| A/Thailand/CU-H3624/2014 | R | N | I | . | . | . | . | . | . | . | S | S | . | . | T | . | S | . | I | . | K | H | S |
| A/Thailand/CU-H3626/2014 | R | N | I | . | . | . | . | . | . | . | S | S | . | . | T | . | S | . | I | . | K | H | S |
| A/Thailand/CU-CB166/2014 | R | N | I | N | . | . | . | . | . | . | T | I | . | . | . | . | S | . | I | . | K | H | S |
| A/Thailand/CU-C4655/2014 | . | . | . | . | . | . | . | . | . | . | N | S | . | . | . | . | S | . | I | . | K | . | S |
| A/Thailand/CU-B10282/2014 | R | N | I | . | . | . | . | . | . | . | S | S | . | . | T | . | S | . | I | . | K | H | S |
| A/Thailand/CU-B10283/2014 | R | N | I | . | . | . | A | S | . | G | N | S | . | S | . | . | S | . | I | . | K | . | S |
| A/Thailand/CU-H3649/2014 | R | N | I | . | . | . | . | . | . | . | S | S | . | Y | T | . | S | . | I | . | K | H | S |
| A/Thailand/CU-B10345/2014 | R | N | I | . | . | . | . | . | . | . | S | S | . | . | T | . | S | . | I | . | K | H | S |
| A/Thailand/CU-B10421/2014 | . | . | . | . | . | . | A | S | . | G | N | S | Q | S | . | . | S | . | I | . | K | . | S |
| A/Thailand/CU-B10422/2014 | R | N | I | . | . | . | A | S | . | G | N | S | . | S | . | . | S | . | I | . | K | . | S |
| A/Thailand/CU-B10509/2014 | R | N | I | . | . | . | A | S | . | G | N | S | . | S | . | . | S | . | I | . | K | . | S |
| A/Thailand/CU-B10520/2014 | R | N | I | . | . | . | . | . | . | . | S | S | . | Y | T | . | S | . | I | . | K | H | S |
| A/Thailand/CU-B10521/2014 | R | N | I | . | . | . | A | S | . | G | N | S | . | S | . | . | S | . | I | . | K | . | S |
| A/Thailand/CU-B10557/2014 | . | N | I | . | . | . | . | . | . | . | S | S | . | Y | T | . | S | . | I | . | K | H | S |
| A/Thailand/CU-H3656/2014 | R | N | I | . | . | . | A | S | . | G | N | S | . | S | . | . | S | . | I | . | K | . | S |
| A/Thailand/CU-B10755/2014 | R | N | I | . | . | . | . | . | . | . | S | S | . | Y | T | . | P | . | I | . | K | H | S |
| A/Thailand/CU-B10792/2014 | R | N | I | . | . | . | . | . | . | . | S | S | . | Y | T | . | S | . | I | . | K | H | S |
| A/Thailand/CU-B10828/2014 | R | N | I | . | . | . | . | . | . | . | N | S | . | . | . | . | P | . | I | . | K | H | S |
| A/Thailand/CU-B10952/2014 | R | N | I | . | . | . | A | S | . | G | N | S | . | S | . | . | S | . | I | . | K | . | S |
| A/Thailand/CU-B10975/2014 | R | N | I | . | . | . | . | . | . | . | S | S | . | Y | T | . | P | . | I | . | K | H | S |
| A/Thailand/CU-B11055/2014 | R | N | I | . | . | . | . | . | . | . | S | S | . | Y | T | . | S | . | I | . | K | H | S |
| A/Thailand/CU-B11065/2014 | R | N | M | . | . | . | . | . | . | . | S | S | . | Y | T | . | P | . | I | . | K | H | S |
| A/Thailand/CU-B11201/2014 | R | N | I | . | . | . | . | . | . | . | S | S | . | Y | T | . | P | . | I | . | K | H | S |
| A/Thailand/CU-B11202/2014 | R | N | I | . | . | . | . | . | . | . | S | S | . | Y | T | . | S | . | I | . | K | H | S |
| A/Thailand/CU-B11284/2014 | R | N | I | . | . | . | . | . | . | . | S | S | . | Y | T | . | P | . | I | . | K | H | S |
| A/Thailand/CU-H3680/2014 | R | N | I | . | . | . | . | . | . | . | S | S | . | Y | T | . | S | . | I | . | K | H | S |
| A/Thailand/CU-B11351/2014 | R | N | I | . | . | . | . | . | . | . | S | S | . | Y | T | . | S | . | I | . | K | H | S |
| A/Thailand/CU-B11367/2014 | R | N | I | . | . | . | . | . | . | . | S | S | . | . | T | . | S | . | I | . | K | H | S |
