# Supplementary material for: Assessing Antigenic Drift of Seasonal Influenza A(H3N2) and A(H1N1)pdm09 Viruses
Source: PLoS One. 2015 Oct 6;10(10):e0139958. doi: 10.1371/journal.pone.0139958 (PMC4594909; doi:10.1371/journal.pone.0139958)
Supplement: S4 Table — (DOCX) [file pone.0139958.s006.docx]

**S4 Table: Amino acid changes observed in the antigenic sites (epitopes A through E) of the HA protein of 81 influenza A(H1N1)pdm09 strains.**

| **Amino acid position** | **69** | **72** | **83** | **94** | **97** | **127** | **143** | **163** | **185** | **190** | **197** | **205** | **216** | **222** | **234** | **245** | **256** | **259** | **267** | **283** |
| --- | --- | --- | --- | --- | --- | --- | --- | --- | --- | --- | --- | --- | --- | --- | --- | --- | --- | --- | --- | --- |
| **Antigenic sites** | **E** | **E** | **E** | **D** |  | **A** | **A** | **D** | **B** | **B** |  | **D** | **D** | **D** |  | **D** |  | **E** | **E** |  |
| **A/California/07/2009** | **S** | **T** | **P** | **D** | **D** | **D** | **S** | **K** | **S** | **S** | **A** | **R** | **I** | **D** | **V** | **T** | **A** | **R** | **I** | **K** |
| A/Thailand/C602/2010 | . | . | S | . | . | . | . | . | . | . | . | . | . | . | . | . | . | . | . | . |
| A/Thailand/CU-B2357/2010 | . | . | S | . | . | . | . | . | . | . | . | . | . | . | . | . | . | . | . | . |
| A/Thailand/CU-B4148/2010 | . | . | S | . | . | . | . | . | T | . | T | . | . | . | . | . | . | . | . | . |
| A/Thailand/CU-B4339/2010 | . | . | S | . | . | . | G | . | T | . | T | . | . | . | . | . | . | . | . | . |
| A/Thailand/CU-C1157/2010 | . | . | S | . | . | . | . | . | T | . | T | . | . | . | . | . | . | . | . | . |
| A/Thailand/CU-H1222/2010 | . | . | S | . | . | . | . | . | . | . | . | . | . | . | . | . | . | . | . | . |
| A/Thailand/CU-H1786/2010 | . | . | S | . | . | . | . | . | . | . | . | . | . | . | . | . | . | . | . | . |
| A/Thailand/CU-H1821/2010 | . | . | S | . | . | . | . | . | . | N | . | . | . | . | . | . | . | . | . | . |
| A/Thailand/CU-H2176/2010 | . | . | S | . | . | . | . | . | . | . | . | . | T | . | . | . | . | . | . | . |
| A/Thailand/CU-H2283/2010 | . | . | S | V | . | . | . | . | T | . | T | . | . | . | . | . | . | . | . | . |
| A/Thailand/CU-H2358/2010 | . | . | S | . | . | . | . | . | T | . | T | . | . | . | . | . | . | . | . | . |
| A/Thailand/CU-H2389/2010 | . | . | S | V | . | . | . | . | T | . | . | . | . | . | . | . | . | . | . | . |
| A/Thailand/CU-H2417/2010 | . | . | S | . | . | . | . | . | . | . | . | . | . | . | . | . | . | . | . | . |
| A/Thailand/CU-H2543/2010 | . | . | S | . | . | . | . | . | T | . | T | . | . | . | . | . | . | . | . | . |
| A/Thailand/CU-H2548/2010 | . | . | S | . | . | . | . | . | T | . | T | . | . | . | . | . | . | . | . | . |
| A/Thailand/CU-H2698/2010 | . | . | S | . | . | . | G | . | T | . | T | . | . | . | . | . | . | . | . | . |
| A/Thailand/H1255/2010 | . | . | S | . | . | . | . | . | . | . | . | . | . | . | . | . | . | . | . | . |
| A/Thailand/H1818/2010 | . | . | S | . | . | . | . | . | . | G | . | . | . | . | . | . | . | . | . | E |
| A/Thailand/CU-H2911/2011 | . | . | S | . | . | . | G | . | T | . | T | . | . | . | . | . | . | . | . | . |
| A/Thailand/CU-B4656/2011 | . | . | S | . | . | E | G | . | T | . | T | . | . | . | . | . | . | . | V | . |
| A/Thailand/CU-B4662/2011 | . | . | S | . | N | . | . | . | . | . | . | K | V | . | . | . | . | . | . | . |
| A/Thailand/CU-B4717/2011 | . | . | S | . | . | . | G | R | T | . | T | . | . | . | . | . | . | . | . | . |
| A/Thailand/CU-B4773/2011 | . | . | S | . | . | . | G | . | T | . | T | . | . | . | . | . | . | . | . | . |
| A/Thailand/CU-B5356/2011 | . | . | S | . | . | E | G | . | T | . | T | . | . | . | . | . | . | . | . | . |
| A/Thailand/CU-B5515/2011 | . | . | S | . | N | E | G | . | T | . | T | K | V | . | . | . | . | . | . | . |
| A/Thailand/CU-B6181/2012 | . | . | S | . | N | . | . | . | T | . | T | . | . | . | . | . | . | . | . | . |
| A/Thailand/CU-B6213/2012 | . | . | S | . | N | . | . | . | T | . | . | K | . | . | . | . | . | . | . | . |
| A/Thailand/CU-B6475/2012 | . | . | S | . | N | . | . | . | T | . | . | . | . | . | . | . | . | . | . | E |
| A/Thailand/CU-B6609/2012 | . | . | S | . | N | . | . | . | T | . | . | . | . | . | . | . | . | . | . | E |
| A/Thailand/CU-B6801/2012 | . | . | S | . | N | . | . | . | T | . | . | . | . | . | I | N | . | . | . | E |
| A/Thailand/CU-B8091/2013 | . | . | S | . | N | . | . | . | T | . | . | . | . | . | I | . | . | . | . | E |
| A/Thailand/CU-B8092/2013 | . | . | S | . | N | . | . | N | T | . | . | . | . | . | I | . | . | . | . | E |
| A/Thailand/CU-B8250/2013 | P | . | S | . | N | . | . | N | T | . | . | . | . | . | I | . | . | . | . | E |
| A/Thailand/CU-A338/2013 | . | . | S | . | N | . | . | Q | T | . | . | . | . | . | . | . | T | . | . | E |
| A/Thailand/CU-B8573/2013 | P | . | S | . | N | . | . | N | T | . | . | . | . | . | I | . | . | . | . | E |
| A/Thailand/CU-B8665/2013 | . | . | S | . | N | . | . | Q | T | . | . | . | . | . | . | . | T | . | . | . |
| A/Thailand/CU-B8730/2013 | . | . | S | . | N | . | . | Q | T | . | . | . | . | . | . | . | T | . | . | E |
| A/Thailand/CU-B8906/2014 | . | . | S | . | N | . | . | Q | T | . | . | . | . | . | . | . | T | . | . | E |
| A/Thailand/CU-B8908/2014 | . | . | S | . | N | . | . | Q | T | . | . | . | . | . | . | . | T | . | . | E |
| A/Thailand/CU-B8981/2014 | . | . | S | . | N | . | . | Q | T | . | . | . | . | . | . | . | T | . | . | E |
| A/Thailand/CU-B8982/2014 | . | . | S | . | N | . | . | Q | T | . | . | . | . | . | . | . | T | . | . | E |
| A/Thailand/CU-B9024/2014 | . | K | S | . | N | . | . | Q | T | . | . | . | . | . | . | . | T | . | . | E |
| A/Thailand/CU-B9037/2014 | . | . | S | . | N | . | . | Q | T | . | . | . | . | . | . | . | T | . | . | E |
| A/Thailand/CU-B9221/2014 | . | . | S | . | N | . | . | Q | T | . | . | . | . | . | . | . | T | . | . | E |
| A/Thailand/CU-B9225/2014 | . | . | S | . | N | . | . | Q | T | . | . | . | . | . | . | . | T | . | . | E |
| A/Thailand/CU-B9433/2014 | . | . | S | . | N | . | . | Q | T | . | . | . | . | . | . | . | T | . | . | E |
| A/Thailand/CU-H3628/2014 | . | . | S | . | N | . | . | Q | T | . | . | . | . | . | . | . | T | . | . | E |
| A/Thailand/CU-H3632/2014 | . | . | S | . | N | . | . | Q | T | . | . | . | . | . | . | . | T | . | . | . |
| A/Thailand/CU-B10114/2014 | . | . | S | . | N | . | G | Q | T | . | . | . | . | . | . | . | T | . | . | E |
| A/Thailand/CU-B10032/2014 | . | . | S | . | N | . | . | Q | T | . | . | . | . | . | . | . | T | . | . | E |
| A/Thailand/CU-B10033/2014 | . | . | S | . | N | . | . | Q | T | . | . | . | . | . | . | . | T | . | . | E |
| A/Thailand/CU-B10044/2014 | . | . | S | . | N | . | . | Q | T | . | . | . | . | . | . | . | T | . | . | E |
| A/Thailand/CU-B10126/2014 | . | . | S | . | N | . | . | Q | T | . | . | . | . | . | . | . | T | . | . | E |
| A/Thailand/CU-B10172/2014 | . | . | S | . | N | . | . | Q | T | . | . | . | . | . | . | . | T | . | . | E |
| A/Thailand/CU-B10174/2014 | . | . | S | . | N | . | . | Q | T | . | . | . | . | . | . | . | T | . | . | E |
| A/Thailand/CU-B10180/2014 | . | . | S | . | N | . | . | Q | T | . | . | . | . | . | . | . | T | . | . | E |
| A/Thailand/CU-B10207/2014 | . | . | S | . | N | . | . | Q | T | . | . | . | . | . | . | . | T | . | . | E |
| A/Thailand/CU-A1058/2014 | . | . | S | . | N | . | . | Q | T | . | . | . | . | E | . | . | T | . | . | E |
| A/Thailand/CU-A1070/2014 | . | . | S | . | N | . | . | Q | T | . | . | . | . | . | . | . | T | . | . | E |
| A/Thailand/CU-C4844/2014 | . | . | S | . | N | . | . | Q | T | . | . | . | . | . | . | . | T | . | . | E |
| A/Thailand/CU-B10185/2014 | . | . | S | . | N | . | . | Q | T | . | . | . | . | . | . | . | T | . | . | E |
| A/Thailand/CU-A1105/2014 | . | . | S | . | N | . | . | Q | T | . | . | . | . | . | . | . | T | . | . | E |
| A/Thailand/CU-B10405/2014 | . | . | S | . | N | . | . | Q | T | . | . | . | . | . | . | . | T | . | . | E |
| A/Thailand/CU-B10578/2014 | . | . | S | . | N | . | . | Q | T | . | . | . | . | . | . | . | T | . | . | E |
| A/Thailand/CU-B10658/2014 | . | . | S | . | N | . | . | Q | T | . | . | . | . | . | . | . | T | . | . | E |
| A/Thailand/CU-B10888/2014 | . | . | S | . | N | . | . | Q | T | . | . | . | . | . | . | . | . | . | . | E |
| A/Thailand/CU-B10909/2014 | . | . | S | . | N | . | . | Q | T | . | . | . | . | . | . | . | T | . | . | . |
| A/Thailand/CU-H3658/2014 | . | . | S | . | N | . | . | Q | T | . | . | . | . | . | . | . | T | . | . | E |
| A/Thailand/CU-C5062/2014 | . | . | S | . | N | . | . | Q | T | . | . | . | . | . | . | . | T | K | . | E |
| A/Thailand/CU-A1205/2014 | . | . | S | . | N | . | . | Q | T | . | . | . | . | . | . | . | T | . | . | E |
| A/Thailand/CU-B11070/2014 | . | . | S | . | N | . | . | Q | T | . | . | . | . | . | . | . | T | . | . | E |
| A/Thailand/CU-C5149/2014 | . | . | S | . | N | . | . | Q | T | . | . | . | . | . | . | . | T | . | . | E |
| A/Thailand/CU-C5169/2014 | . | . | S | . | N | . | . | Q | T | . | . | . | . | . | . | . | T | . | . | E |
| A/Thailand/CU-A1282/2014 | . | . | S | . | N | . | . | Q | T | . | . | . | . | . | . | . | T | . | . | E |
| A/Thailand/CU-B11233/2014 | . | . | S | . | N | . | . | Q | T | . | . | . | . | . | . | . | T | . | . | E |
| A/Thailand/CU-B11291/2014 | . | . | S | . | N | . | . | Q | T | . | . | . | . | . | . | . | T | . | . | E |
| A/Thailand/CU-C5263/2014 | . | . | S | . | N | . | . | Q | T | . | . | . | . | . | . | . | I | . | . | E |
| A/Thailand/CU-A1344/2014 | . | . | S | . | N | . | . | Q | T | . | . | . | . | . | . | . | T | . | . | E |
| A/Thailand/CU-B11363/2014 | . | . | S | . | N | . | . | Q | T | . | . | . | . | . | . | . | T | . | . | E |
| A/Thailand/CU-B11375/2014 | . | . | S | . | N | . | . | Q | T | . | . | . | . | . | . | . | T | . | . | E |
| A/Thailand/CU-B11422/2014 | . | . | S | . | N | . | . | Q | T | . | . | . | . | . | . | . | T | . | . | E |
